# N-myc downstream–regulated gene 1 can promote vasculogenic mimicry and angiogenesis in urothelial carcinoma

**DOI:** 10.1007/s00428-024-03793-w

**Published:** 2024-04-02

**Authors:** Ereny Kamal Louis, Islam F. Abdelkawi, Abeer Refaiy, Asmaa M. Ahmed

**Affiliations:** 1 Pathology Department, Faculty of Medicine, Assiut University, Assiut, Egypt; 2 Assiut University Urology Hospital,Faculty of Medicine, Assiut University, Assiut, Egypt

**Keywords:** Urothelial carcinoma, NDRG1, Vasculogenic mimicry, MVD

## Abstract

Urothelial carcinoma (UC) of the bladder is a common cause of cancer-related death worldwide. Vasculogenic mimicry (VM) is a process by which the malignant cells can generate vascular-like structures formed of periodic acid–Schiff (PAS) positive/CD31 negative extracellular matrix independent of angiogenesis and thus promotes tumor progression. N-myc downstream–regulated gene 1 (NDRG1) is a protein that can modulate tumor angiogenesis; however, its role in regulating tumor angiogenesis and VM formation has not been previously investigated in UC. This study aims to evaluate the role of intra-tumor microvessel density (MVD) (as a surrogate measure of angiogenesis), VM, and NDRG1 in UC and their correlation with different clinicopathologic features, then assess the correlation between them in UC. Sixty specimens of UC of the bladder were included. PAS-CD31 immunohistochemical double staining method was used to evaluate the intra-tumor MVD and VM. Immunohistochemical expression of NDRG1 was also examined. VM and NDRG1 expression were detected in 41.7% and 83.3% of UC specimens respectively. The mean of intra-tumor MVD, VM area, and NDRG1 was significantly higher in tumors with higher grade, lymphovascular invasion, and higher T stage. NDRG1 expression was positively correlated with MVD and VM. We can suggest that MVD, VM, and NDRG1 may serve as poor prognostic markers for UC. The positive correlation between NDRG1 and both MVD and VM may provide the first evidence that NDRG1 can induce tumor angiogenesis and VM in UC which may offer a novel pathway for further therapeutic strategies.

## Introduction

Globally, bladder cancer is the 7th most common cancer and the 13th most common cause of cancer-related death [1]. Urothelial carcinoma (UC) is the most common histologic type, constituting about 90% of all bladder cancers [2]. The majority of UC initially arise as non-muscle invasive (NMI) with a high rate of recurrence after transurethral resection and a subgroup of high-risk lesions frequently progresses to invasive forms [2, 3]. Conversely, 20–30% of UC originally present as muscle-invasive disease with high rates of metastasis [2, 3]. Unfortunately, there is still a significant percentage of UC patients with poor prognosis and lower survival after the current therapeutic strategies (radical cystectomy, radiotherapy, chemotherapy, and immunotherapy) [4]. Therefore, clarifying the mechanism of progression, invasion, and spread of UC has become a key focus of research to find new targets for therapy [4].

Tumor microcirculation plays a major role in growth and dissemination of cancer cells [5]. Tumor angiogenesis has been known to be the sole method by which tumors acquire their blood supply. It is characterized by true vasculogenesis in which de novo endothelial cell–lined vessels are formed and induced by specific factors secreted by tumor cells and the surrounding microenvironment [5]. The angiogenic potentials of tumors are assessed by measuring the microvessel density (MVD) using endothelial markers such as CD31 [6]. However, several studies demonstrated that anti-angiogenic drugs which induce endothelial cell apoptosis have a little effect in many tumors suggesting that other novel tumor microcirculation patterns may exist in these neoplasms [5, 7].

Vasculogenic mimicry (VM) is a process by which the malignant cells can generate vascular-like structures independent of angiogenesis [8]. It was first described in melanoma cells as non-endothelial cell–lined microcirculatory channels formed of periodic acid–Schiff (PAS) positive extracellular matrix material (ECM) and lined externally by tumor cells [8]. These channels can directly connect with the surrounding blood vessels to conduct fluid, nutrients, and red blood cells to the tumor cells [8]. Morphologically, two types of VM have been described (tubular and patterned matrix types) [9]. The tubular type is characterized by non-endothelial cell–lined tubes that contain red blood cells (RBCs) resembling blood vessels [9]. The patterned matrix type is formed of PAS-positive ECM arranged in arcs and loops surrounding packets of tumor cells [9]. This patterned matrix type may branch out into smaller arcs with hollow channels that contain RBCs [10]. Based on these characters, VM can be distinguished using histochemical and immunohistochemical double staining. While VM is negative for endothelial markers (CD34- or CD31-) and positive for periodic acid–Schiff (PAS) stain, classic blood vessels are double positive for endothelial markers and PAS stain [10]. The presence of VM has been associated with poor prognosis, low survival, and therapeutic resistance in several tumors [11]. Despite the great advances in identifying VM in the last few years, the key mechanisms of VM formation are not fully elucidated [11].

N-myc downstream–regulated gene 1 (NDRG1) is a protein encoded by a gene located on chromosome 8q24.22 and it is a member of the human NDRG family [12]. It participates in regulating certain biologic processes including cellular growth, differentiation, stress, and hormonal responses [12]. Recently, several researches have pointed to the role of NDRG1 in the tumorgenesis [13, 14]. Interestingly, it has been reported that NDRG1 can modulate tumor growth and angiogenesis through regulating the angiogenic “on- or off-switch” of the tumor stroma [15, 16]. It was found that NDRG1 can promote tumor angiogenesis and growth in certain cancers as lung carcinomas [15] while suppressing angiogenesis [16] and VM [17] in others. However, the role of NDRG1 and its correlation with tumor angiogenesis and VM in urothelial carcinoma have not been extensively studied and remain to be further clarified.

This study aimed at assessing the role of intra-tumor MVD (as a surrogate measure of angiogenesis), VM, and NDRG1 in urothelial carcinomas and their correlation with different clinicopathologic features, then assessing the correlation between VM, MVD, and NDRG1 in urothelial carcinomas.

## Material and methods

### Specimens

This study was approved by the Institutional Ethics and Research Committee of the Faculty of Medicine, Assiut University, Assiut, Egypt (IRB no: 17100939). This is a retrospective study that included formalin-fixed paraffin-embedded blocks of sixty specimens of urothelial carcinoma of the bladder. They include 38 transurethral resection (TUR) specimens and 22 cystectomy specimens. The blocks were retrieved from the archives of Surgical Pathology Laboratory at Assiut University Hospital, Faculty of Medicine, Assiut University (in the period between 2018 and 2021). The clinical and radiologic data included patient’s age, gender, and tumor size that were obtained from the hospital medical records. Hematoxylin and eosin–stained slides of urothelial carcinoma were reexamined for detailed histopathologic features including histologic grade (according to the WHO 5^th^ edition (2022) [18]), staging (according to American Joint Committee on Cancer, 8th edition [19]), presence or absence of lymphovascular invasion, lymph node metastasis, necrosis, and associated bilharzial cystitis.

### Immunohistochemical staining of CD31 and NDRG1

Deparaffinization and rehydration of 4-μm-thick sections were first performed followed by blocking the endogenous peroxidase activity by H2O2 (3%). Antigen retrieval was performed by immersing the sections in 10 mmol/l citrate buffer at pH 6.0 and heating at 80 °C in a microwave for 15 min. Then, the sections were incubated with the primary antibodies (CD31: Mouse monoclonal antibody (Clone JC/70A), Catalog number A00009-C-IFU-RUO, Scy Tek, USA, dilution 1:100 and NDRG1: Rabbit monoclonal antibody, Catalog number A4050, dilution 1:100, ABclonal Technology, USA) overnight at room temperature (37 °C). Secondary staining kits were used according to the manufacturer’s instructions (Thermoscientific Corporation, Fremont, CA, USA).

### Periodic acid–Schiff-CD31 double staining for detection of vasculogenic mimicry

After incubation of the sections by the primary antibody (CD31) and applying the secondary antibodies and DAB reaction, the sections were exposed to 1% sodium periodate for 10 min. Then, the sections were rinsed with distilled water for 5 min, and incubated with 0.5% periodic acid solution for 10 min. After that, the sections were treated with Schiff’s reagent for 15 min in the dark and washed with distilled water for 5 min. Then, the sections were counterstained with hematoxylin [20].

### Immunohistochemical evaluation

CD31 was used to count the intra-tumor MVD. CD31 cytoplasmic staining in endothelial cells was considered positive. The sections were initially examined at low magnification (×100) to find the most vascularized areas (hot spots). After that, five images at high-power field (×400) of these hot spot areas were captured using Toup-Cam Full HD digital camera (model number: XCAM1080PHB) attached to Olympus Microscope BX43. Then, the number of CD31 positive vessels was counted using open-access ImageJ and the mean value was calculated [21].

In order to assess the VM area, the CD31/PAS stained slides were scanned by low power to identify areas which are positive for VM, and then five images at high-power field (×400) were captured. The VM area was recorded in these five high-power fields (400×) using open-access ImageJ and then averaged [22].

For NDRG1, cytoplasmic and/or membranous staining was considered positive [23]. Histological score *(H* score) method was used for evaluation. The percentage of positive cells (0–100) was multiplied with the staining intensity (1: weak, 2: moderate, 3: strong) to give an *H* score between 0 and 300 [23].

### Statistical analysis

All analyses were performed by the statistical software package SPSS (Statistical Package for Social Science, Inc. version 16, Chicago). The Kolmogorov-Smirnov test was used to test the normality. The Mann-Whitney and Kruskal-Wallis tests were used to compare the mean of NDRG1, VM area, and MVD in urothelial carcinoma in relation to different clinicopathological features. The Spearman correlation coefficient was used to investigate the correlation between NDRG1 expression, VM, and MVD in urothelial carcinoma. *p* value of <0.05 was regarded as statistically significant.

## Results

### Clinicopathological features of the studied cases

The mean age of urothelial carcinoma patients was 60.53± 10.53 (ranged from 35 to 83 years ). The clinicopathological features are summarized in Table 1.

**Table 1:** Clinicopathological characteristics of the studied cases (*n* = 60)

Variables	No of cases	Percentage
*Age*		
≤ 60	27	45%
> 60	33	55%
*Gender*		
Male	54	90%
Female	6	10%
*Size of tumor*		
≤3 cm	10	16.7%
>3 cm	50	83.3%
*Histological variant*		
Conventional urothelial carcinoma	45	75%
Urothelial carcinoma with squamous differentiation	11	18.3%
Urothelial carcinoma with glandular differentiation	2	3.3%
Micropapillary variant	1	1.7%
Microcystic variant	1	1.7%
*Grade of tumor*		
Low grade	22	36.7%
High grade	38	63.3%
*Lymphovascular invasion*		
Present	18	30%
Absent	42	70%
*Lymph node metastasis*		
Present	8	13.3%
Absent	14	23.3%
Can’t be assessed (pNX)	38	63.3%
*Necrosis*		
Present	20	33.3%
Absent	40	66.7%
*Bilharziasis*		
Present	7	11.7%
Absent	53	88.3%
*pT stage*		
pTa	8	13.3%
pT1	14	23.3%
pT2	18	30%
pT3	17	28.3%
pT4	3	5%
*pN stage*		
pNX	38	63.3%
pN0	14	23.3%
pN1	5	8.3%
pN2	3	5%

### Immunohistochemical results

#### Evaluation of intra-tumor microvessel density in urothelial carcinomas and its correlation with the clinicopathologic features

**Fig. 1:**
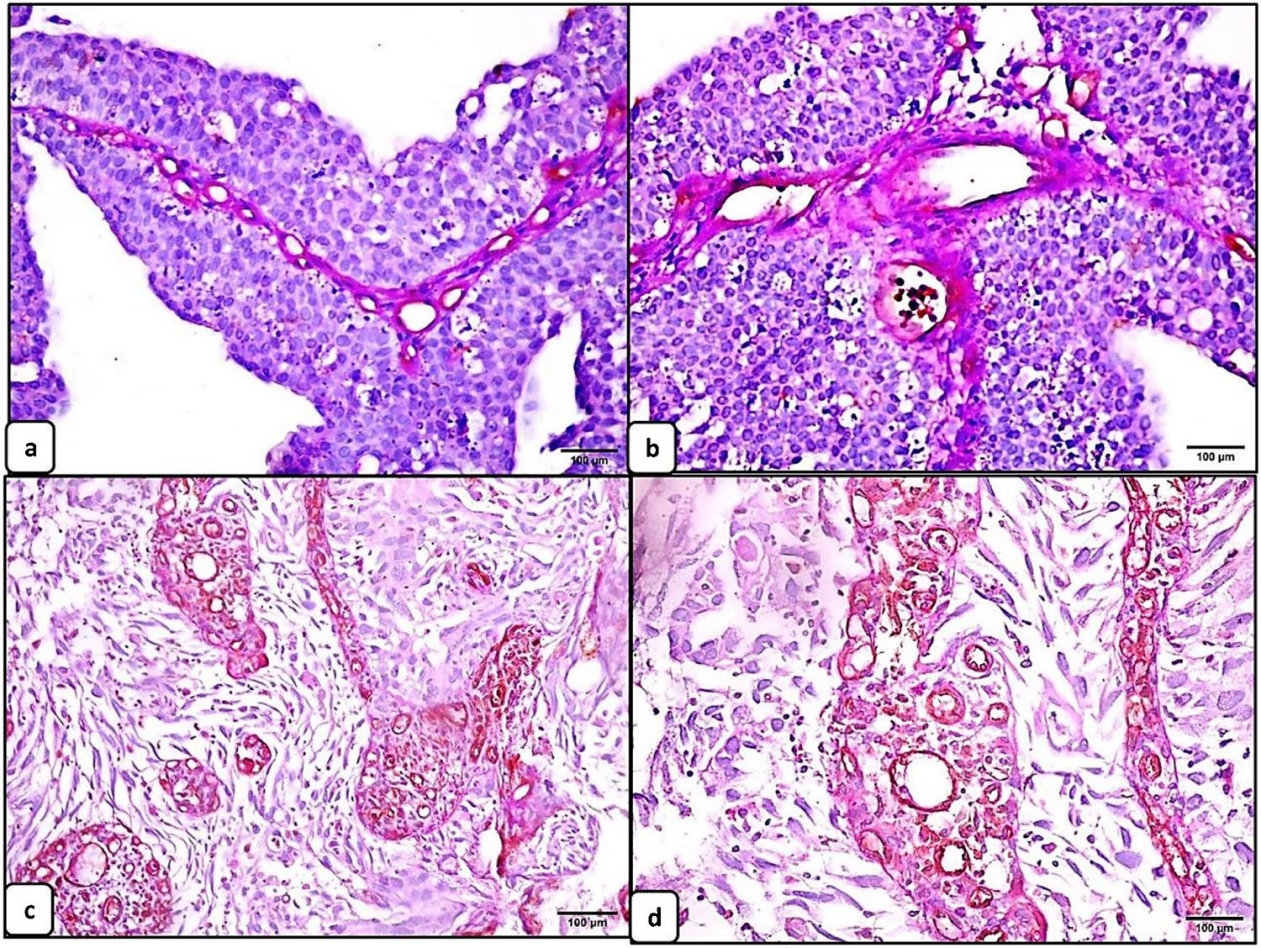
Microvessel density in urothelial carcinoma (scale bar 100 μm). Low microvessel density in low-grade urothelial carcinoma (**a**, ×200 and **b**, ×400). High microvessel density in high-grade urothelial carcinoma (**c**, ×200 and **d**, ×400)

PAS +ve /CD31 +ve endothelial cell–lined blood vessels were counted in five high-power fields (×400) and averaged. The intra-tumor MVD was significantly higher in urothelial carcinomas with higher grade (median = 14.80; mean ±SD=15.58±4.84)(*p* =0.001) (Fig. 1), lymphovascular invasion (median =18.70; mean ±SD= 18.60±4.41) (*p* <0.0001), and higher T stage (median = 22.60; mean ±SD= 20.60 ± 4.91) (*p* <0.0001). However, insignificant difference was detected between the intra-tumor MVD and the remaining clinicopathologic features (Table 2).

**Table 2:** Relationship between MVD, VM and NDRG1 expression with the clinicopathological features of the studied cases

Variables	Mean of microvessel density ± SD	*p* value	Mean of vasculogenic mimicry area ± SD	*p* value	Mean of NDRG1 H score ± SD	*p* value
*Age*		0.345		0.219		0.438
≤ 60	13.21±4.40		19.22±20.17		138.51±104.53	
˃ 60	15.01±5.42		12.86±19.45		157.27±95.86	
*Gender*		0.521		0.296		0.111
Male	14.27±4.94		16.59±19.90		142.96±100.23	
Female	13.53±6.21		7.97±19.52		201.66±80.35	
*Size of tumor*		0.366		0.215		0.228
≤3 cm	13.30±5.14		8.75±18.48		112±110.33	
>3 cm	14.38±5.04		17.12±20.01		156.20±96.63	
*Grade of tumor*		0.001*		<0.0001*		<0.0001*
Low grade	11.80±4.48		0		83.18±89.03	
High grade	15.58±4.84		24.83±19.93		186.84±84.98	
*Lymphovascular invasion*		<0.0001*		0.009*		0.006*
Present	18.60±4.41		25.93±19.08		201.11±103.74	
Absent	12.31±4.01		11.35±18.75		126.42±89.73	
*Lymph node metastasis*		0.101		0.579		0.681
Present	14.32±0.84		27.63±19.22		206.25±104.18	
Absent	19.15±5.83		22.68±19.57		194.28±96.61	
*Necrosis*		0.187		0.066		0.474
Present	15.31±5.31		22.06±16.02		162.50±102.59	
Absent	13.64±4.84		12.56±21.00		142±98.43	
*Bilharziasis*		0.205		0.057		0.055
Present	16.62±5.81		28.80±17.04		218.57±49.47	
Absent	13.87±4.88		13.99±19.70		139.62±100.97	
*pT stage*		<0.0001*		<0.0001*		0.002*
pTa	9.82±2.74		0		50±47.80	
pT1	12.92±4.96		0		102.14±102.52	
pT2	13.04±2.79		24.42±21.30		173.88±63.44	
pT3	17.40±5.24		23.85±19.98		190±107.47	
pT4	20.60±4.91		32.77±13.92		246.66±5.77	
*pN stage*		0.424		0.063		0.515
pN0	19.15±5.83		22.68±19.57		194.28±96.61	
pN1	14.44±0.89		16.88±15.94		176±125.81	
pN2	14.13±0.90		45.57±3.50		256.66±15.27	

*Significant (Mann-Whitney and Kruskal-Wallis tests) (*p* <0.05)

#### Evaluation of vasculogenic mimicry in urothelial carcinomas and its correlation with the clinicopathologic features

**Fig. 2:**
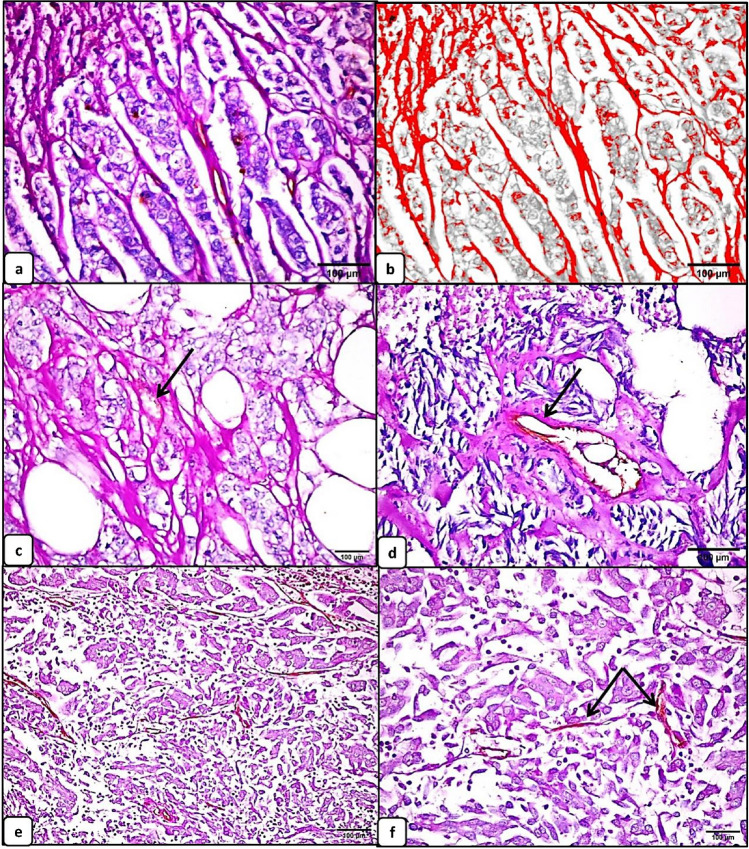
Vasculogenic mimicry (VM) by periodic acid–Schiff (PAS)-CD31 double staining in urothelial carcinoma (scale bar 100 μm). PAS +ve /CD31 −ve patterned matrix type of vasculogenic mimicry arranged in back-to-back loops and arches that surrounded packets of tumor cells (arrow) (**a**, ×400). Vasculogenic mimicry by ImageJ analysis; the patterns have been automatically detected, highlighted, and quantified (**b**, ×400). Red blood cells are focally noted within the lumen of PAS +ve /CD31 −ve patterned matrix type of vasculogenic mimicry (arrow) (**c**, ×400). VM structures are interconnected with CD31+ endothelial-lined blood vessels in some areas (**d**, ×400). Urothelial carcinoma negative for vasculogenic mimicry and contains only CD31+ endothelial-lined blood vessels (arrows) (**e**, ×200 and **f**, ×400)

Vasculogenic mimicry (VM) was detected in 25 specimens (41.7%) of urothelial carcinoma while the remaining 35 specimens were negative. All the positive specimens were high grade. The patterned matrix type of VM was detected in all positive specimens. This pattern was seen as PAS +ve /CD31 −ve networks, back-to-back loops, and arches that surrounded packets of tumor cells. In some areas, these PAS +ve /CD31 −ve patterns were interconnected with CD31+ endothelial cell–lined blood vessels. Red blood cells were focally noted within the lumen of these PAS +ve /CD31 −ve patterns (Fig. 2).

The VM area was significantly higher in urothelial carcinomas with higher grade (median = 30.87; mean ±SD= 24.83±19.93) (*p* <0.0001), lymphovascular invasion (median = 28.32; mean ±SD= 25.93±19.08 ) (*p* =0.009), and higher pathologic T stage (median = 31.34; mean ±SD= 32.77± 13.92) (*p* <0.0001), while the difference between the VM area and the other clinicopathologic parameters was not significant (Table 2).

#### Immunohistochemical expression of NDRG1 and its correlation with the clinicopathologic features

**Fig. 3:**
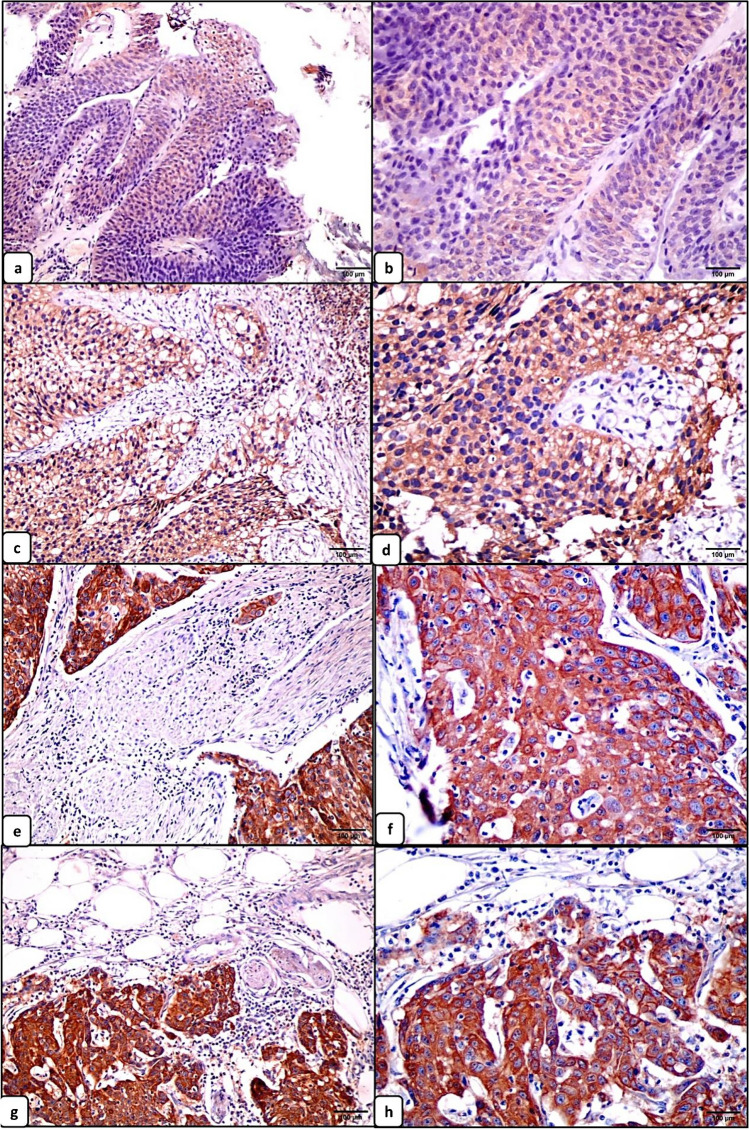
Immunohistochemical expression of NDRG1 in urothelial carcinoma (scale bar 100 μm). Weak expression of NDRG1 in low-grade non-invasive urothelial carcinoma (**a**, ×200 and **b**, ×400). Moderate expression of NDRG1 in low-grade urothelial carcinoma infiltrating the lamina propria (**c**, ×200 and **d**, ×400). Strong expression of NDRG1 in high-grade urothelial carcinoma infiltrating the muscle layer (**e**, ×200 and **f**, ×400). Strong expression of NDRG1 in high-grade urothelial carcinoma infiltrating the perivesical fat (**g**, ×200 and **h**, ×400)

Positive NDRG1 expression was detected in 50/60 (83.3%) of urothelial carcinoma specimens. Its expression was significantly higher in tumors with higher grade (median = 200; mean±SD = 186.84 ±84.98) (*p*<0.0001), positive lymphovascular invasion (median =245; mean ±SD= 201.11±103.74) (*p* =0.006), and higher T stage (median = 250; mean ±SD= 246.66±5.77 ) (*p*= 0.002) (Fig. 3). However, no significant difference was detected between the NDRG1 expression and the remaining clinicopathologic parameters (Table 2).

#### Correlation between microvessel density, vasculogenic mimicry, and NDRG1 expression

There was a statistically significant positive correlation between the expression of NDRG1 and microvessel density (*p* = 0.011, *r* = 0.325). Also, statistically significant positive correlation between expression of NDRG1 and VM area was found (*p* = 0.001, *r* = 0.431), while no statistically significant correlation between VM area and microvessel density was present (*p* = 0.076, *r* = 0.231).

## Discussion

Although urothelial carcinomas of the bladder may be cured by either surgical or non-surgical therapeutic options, high rates of metastasis and/or recurrence still constitute the most common cause of death in these patients and represent the major challenges in clinical management [3]. Given the relapsing character of this disease, it is obvious that there is an urgent need to find efficient molecules and signaling pathways implicated in the molecular pathogenesis and progression of UC [4]. This might offer novel markers for personalized therapy and improved prognosis [4]. This study aimed to investigate the role of intra-tumor MVD (as a surrogate measure of angiogenesis), VM, and NDRG1 in urothelial carcinomas.

Angiogenesis is a multistep dynamic process, which is characterized by the proliferation, migration, and differentiation of endothelial cells into new capillaries. MVD is used to evaluate tumor vasculature using endothelial markers [24]. In the current study, high intra-tumor MVD was associated with higher tumor grade, stage, and lymphovascular invasion. This finding is in agreement with previous studies in urothelial carcinoma [24, 25] suggesting that assessment of tumor vascularity may be useful in predicting patients’ prognosis and selecting those who will benefit from anti-angiogenic therapies [25]. It has been reported that the initiation of angiogenesis occurs when the proangiogenic factors (e.g., vascular endothelial growth factor) surpass anti-angiogenic factors which result in progressive tumor growth [26]. Interestingly, it has been recognized that the newly formed vasculature not only transports nutrients and oxygen but also provides a route for cancer cells to enter the systemic circulation [26]. Tumor vessels differ from normal blood vessels by fenestrated lining with endothelial gaps and lacking the pericyte layer which make them highly permeable. All of this facilitates cancer cell dissemination and metastasis [26].

Despite the ever-growing list of anti-angiogenic drugs, their effect is limited, only provide short-term relief from tumor growth before resistance occurs [27]. This limited efficacy may be explained by the ability of some tumors to employ alternative sources for neoplastic perfusion [27]. Vasculogenic mimicry is a process by which tumor cells can create vessel-like structures without contribution of endothelial cells [11]. Consistent with the results of the present work, vasculogenic mimicry was detected in several tumors including urothelial carcinoma [7, 28], melanoma [22], medulloblastoma [10], and gliomas [29]. It has been described that cancer stem cells support the formation of ECM with subsequent induction of VM in tumor tissues to ensure additional route for sufficient blood supply and nutrients independent of tumor angiogenesis [11].

The patterned matrix type of VM was detected in all positive specimens in this study which appeared as PAS +ve /CD31 −ve back-to-back loops and arches that surrounded packets of tumor cells, contained RBCs, and interconnected with CD31+ endothelial cell–lined blood vessels in some areas. In support, Wang et al. reported that PAS-positive patterns in medulloblastoma constitute a part of the tumor microcirculation, based on the following morphological criteria: CD34 −ve/ PAS +ve patterns that branched out into smaller arches with hollow channels contained RBCs with direct communication with blood vessels [10].

In accordance with previous studies [9, 30], the patterned matrix type of VM was correlated with poor prognostic factors as high tumor grade [9, 30], stage [9], and lymphovascular invasion [9]. The patterned matrix type of VM has been suggested to be a characteristic of highly invasive tumors as it could provide a greater surface area for diffusion and support tumor growth in early stages of tumorgenesis [10]. Although this pattern appears as PAS +ve thin lines by light microscopy, Frenkel et al. described that these structures appeared as hollow channels with blood circulation in their lumens by laser scanning confocal angiography in a choroidal melanoma [31]. Moreover, Manarang JC et al. described that highly aggressive melanoma cells cultured in 3 dimensional matrix gel induced the formation of VM with characteristic looping patterns that can transmit fluids, while less aggressive cells did not produce these VM patterns when grown on similar conditions [32]. Interestingly, it has been also suggested that VM not only promotes tumor growth but also facilitate lymphovascular invasion. Shirakawa et al. reported the presence of a VM-angiogenesis junction by transmission electron microscopy in which cancer cells within the tumor-lined vascular channels can easily transfer into endothelial-lined blood vessels, facilitating tumor cell metastasis [33].

On the contrary to the previous findings, Li B et al. study did not find significant association between VM and the clinicopathologic features of urothelial carcinoma patients [7].

Consistent with previous studies [3, 34], the current results demonstrated that over-expression of NDRG1 was correlated with higher tumor grade [34], stage [3], and lymphovascular invasion [34]. The role of NDRG1 in urothelial carcinoma has not been extensively investigated. Li A et al. found that higher NDRG1 expression was correlated with higher tumor stages and increased cellular proliferation and invasiveness of urothelial carcinoma through promotion of several epithelial mesenchymal transition (EMT) transcription factors [3]. However, the opposite results were reported in gastric carcinoma where NDRG1 was suggested to be a tumor suppressor protein through inhibition of multiple oncogenic signal pathways [35]. NDRG1 down-regulation was associated with higher tumor grade, stage, and lymphatic invasion [35]. Therefore, NDRG1 can exert contradictory role in cancer depending primarily on the tissue type affected [36].

NDRG1 can promote tumor growth through up-regulation of several proteins involved in angiogenesis (e.g., vascular endothelial growth factor and Interleukin-1α) [36, 37]. Similar to the current results, Murakami et al. demonstrated a significant positive correlation between the number of newly formed vessels and NDRG1 over-expression in gastric carcinoma [37]. In contrast, Maruyama et al. described a significant negative correlation between NDRG1 expression and MVD in pancreatic cancer as NDRG1 can reduce the expression of angiogenic factors such as VEGF [38]. Thus, NDRG1 could play a major role in the angiogenic on- or off-switch of tumor stroma [38].

No previous study assessed the correlation between NDRG1 and MVD in urothelial carcinoma. So, further studies are required to understand the underlying mechanism of how NDRG1 expression can modulate the expression of angiogenesis-related proteins.

This is the first study which reported a significant positive correlation between NDRG1 expression and vasculogenic mimicry. There is an increasing evidence that EMT is an important promoter for VM formation in malignant tumors [39]. Many transcriptional factors involved in EMT, such as Slug, bone morphogenetic protein 4, and Snail2, can induce VM formation [39]. Interestingly, a recent study reported that NDRG1 over-expression promoted EMT in bladder cancer through up-regulation of EMT-related transcription factors including Twist1 and Snail2 [3]. Thus, we can suggest that NDRG1 can promote VM formation by its role in induction of EMT. This might explain the positive association between NDRG1 and VM in the current study. On the contrary, NDRG1 can suppress VM formation in gastric carcinoma through inhibition of EMT [17]. This contradictory role of NDRG1 on tumor angiogenesis and VM formation in different cancer types confirming its tissue-specific pleiotropic role in cancer. However, the underlying mechanisms that trigger NDRG1 pleiotropy are still unknown and required further investigations [36].

Regarding the correlation between VM and MVD, no significant correlation between them was detected in the present study. In agreement, Wu Y. et al. found no difference in MVD between VM-positive group and VM-negative group in renal cell carcinoma [40]. Interestingly, Wang et al. reported a negative correlation between MVD and VM in medulloblastoma [10]. They reported that the MVD counts in the VM-positive group were significantly less than in the VM-negative group, which is indirect evidence of the blood supply function of VM [10]. Moreover, currently widespread used anti-angiogenic drugs may have no effect on VM or even inducing VM formation when blood vessels are destroyed leaving a hypoxic environment [29]. This may suggest that VM can provide neoplastic perfusion independent on angiogenesis. Therefore, newly developed drugs based on anti-angiogenic strategies must take both anti-angiogenic and anti-VM treatment into serious consideration [10].

In conclusion, the current results suggested that MVD, VM, and NDRG1 expression may serve as poor prognostic markers for urothelial carcinoma. To our knowledge, this study provided the first evidence that higher NDRG1 expression can induce tumor angiogenesis and vasculogenic mimicry in urothelial carcinoma which may provide a novel pathway for further therapeutic strategies. However, this study had some limitations as a relatively small sample size without addressing the role of MVD, VM, and NDRG1 in patients’ survival. Therefore, further prospective studies using larger sample size are recommended to evaluate the effects of MVD, VM, and NDRG1 on the survival of urothelial carcinoma patients. Also, the exact mechanism by which NDRG1 can affect tumor angiogenesis and VM formation in urothelial carcinoma requires further investigations.

## Data Availability

The data that support the findings of this study are available from the corresponding author upon reasonable request.
